# High-resolution whole-heart 3D T_2_ mapping can assess tissue heterogeneity of chronic MI in swine

**DOI:** 10.1186/1532-429X-17-S1-P242

**Published:** 2015-02-03

**Authors:** Haiyan Ding, Karl H Schuleri, Michael Schär, Henry R Halperin, Roy Beinart, Muz M Zviman, Daniel A Herzka

**Affiliations:** Center for Biomedical Imaging Research, Department of Biomedical Engineering, Tsinghua University, Beijing, China; Department of Biomedical Engineering, Johns Hopkins School of Medicine, Baltimore, MD USA; Department of Medicine, Cardiology, Johns Hopkins School of Medicine, Baltimore, MD USA; Department of Radiology, Mercy Fitzgerald Hospital, Darby, PA USA; Russell H. Morgan Department of Radiology and Radiological Science, Johns Hopkins School of Medicine, Baltimore, MD USA; Heart Institute, Sheba Medical Center, Tel Aviv University, Ramat Gan, Israel

## Background

Remodeling of myocardium after infarction (MI) is linked to ventricular arrhythmias. [1] It has been demonstrated that the presence of scar containing isthmuses of viable myocardium resulting in a heterogeneous zone (HZ) with altered conduction properties which may be part of the critical substrate for post-MI ventricular tachycardia. [2, 3] Late gadolinium-enhanced (LGE) imaging is used for MI visualization, clearly depicting infarct size and transmurality due to the excellent contrast achieved between scar and viable tissue. However, with LGE uncertainty can be introduced by contrast agent kinetics. [4] Furthermore, LGE can lack information on tissue heterogeneity beyond "gray" areas that result from partial volume averaging and are assumed to be representative of the HZ. Conversely, scar tissue also exhibits increased T_2_, as fibrosis, primarily composed of collagen, increases interstitial water per unit volume. [5] Hence, direct and quantitative measurement of T_2_ relaxation time may be a feasible alternative for delineating viable myocardium and fibrosis with the additional benefit of depicting tissue heterogeneity.

### Hypothesis

High-resolution whole-heart 3D T_2_ mapping can assess tissue heterogeneity of chronic MI without contrast agents.

## Methods

MI was induced in swine (N=3) by 2 hr balloon occlusion of the LAD after the first diagonal. MRI was carried out 4-6 months post MI (Achieva TX, Philips). Whole-heart 3D respiratory navigator-gated T_2_-mapping [6] was performed. Serial gadolinium-enhanced images using PSIR [7] were acquired at 3, 5, 10 and 20 min post infusion using 0.2 mmol/kg Gadolinium-based contrast (Magnevist). After final MRI, hearts were excised, imaged *ex vivo*, and post-mortem pathology and histology (H&E, Masson's Trichrome) were obtained.

## Results

**Figure 1:**
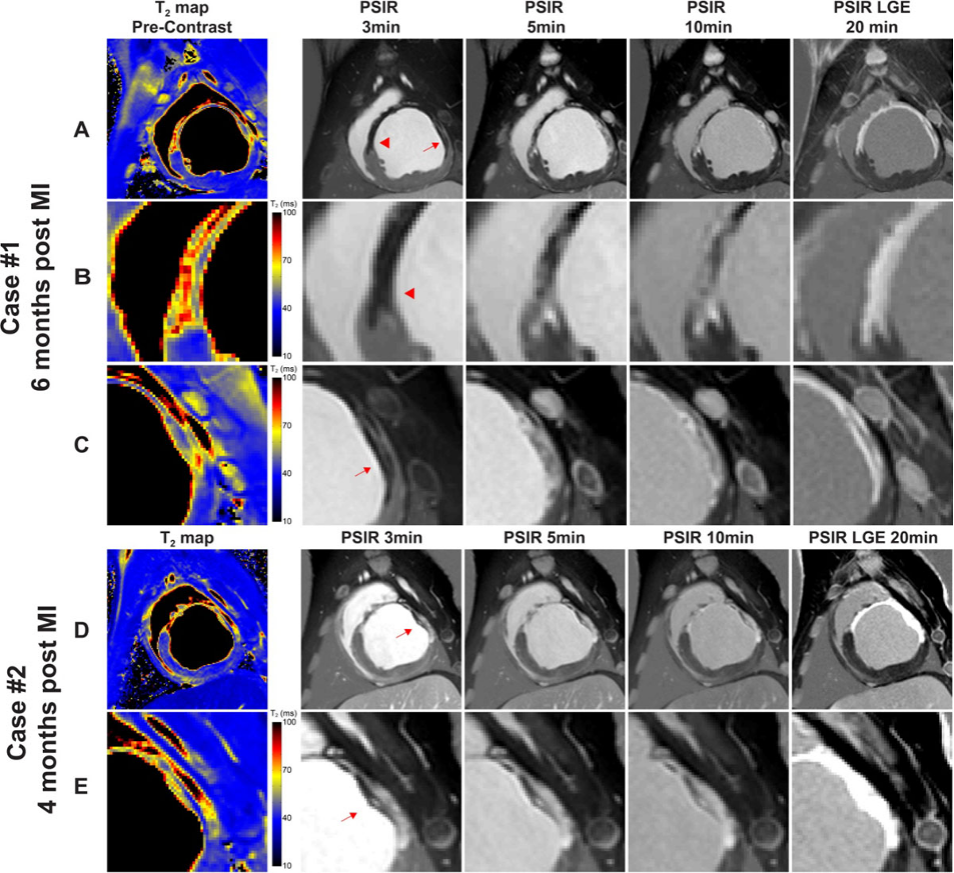
Matched representative SAX images from two swine 6 months post MI (Case #1, A-C) and 4 months post MI (Case #2, D-E). Image series A and D includes post-contrast PSIR and pre-contrast T_2_ map. The borders of the infarct (arrowhead and arrows) were magnified in series B, C and E. The contrast kinetics revealed by the PSIR images (3-10 min post) demonstrates that the infarct is in effect heterogeneous, while the LGE (PSIR 20 min) failed to visualize the heterogeneity. The pre-contrast T_2_ map identified the infarct and co-localized with LGE excellently, and preserved the tissue characteristics very well.

**Figure 2:**
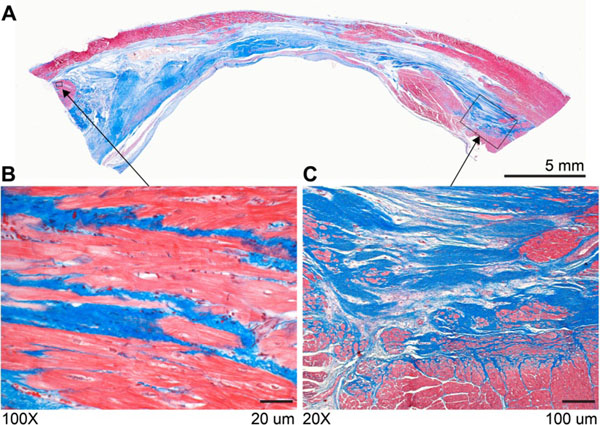
A. Masson's Trichrome staining of MI after 6 months (Case #1 in Figure 1) depicts viable myocardium in red and fibrotic tissue in blue. B. Heterogeneous boundary of infarct demonstrates the mixture of viable tissue and fibrosis. C. Magnification of the isthmuses of viable myocardium mingled with fibrosis.

T_2_ maps showed excellent correlation with the myocardial distribution of infarct as evidenced by significantly and variably elevated T_2_ and the correlation with hyper-enhanced infarct area from LGE (PSIR 20 min post). Heterogeneity in the changes in signal intensity as a result of contrast agent kinetics was clearly visible on serial PSIR (3 - 20 min). LGE described the infarcted area uniformly, regardless of tissue heterogeneity. High spatial resolution T_2_ mapping enabled heterogeneity detection in and around the infarct area (Fig 1B,C,E). Histological images in Figure 2 shows that collagen deposition penetrated into normal myocardium at the border zones of the MI, yet thin layers of viable myocardium remain within the infarct even after 6 months.

## Conclusions

Images demonstrate that high-resolution 3D myocardial T_2_ mapping has the potential to noninvasively characterize chronic MI size, transmurality, and heterogeneity without exogenous contrast agents, providing an alternative for HZ determination beyond the traditional "gray" zone.

## Funding

Funded in part by the American Heart Association -11SDG5280025.
